# Explosion-related deaths: An overview on forensic evaluation and implications

**DOI:** 10.1007/s12024-021-00383-z

**Published:** 2021-07-01

**Authors:** Nicola Galante, Lorenzo Franceschetti, Sara Del Sordo, Michelangelo Bruno Casali, Umberto Genovese

**Affiliations:** Dipartimento Di Scienze Biomediche Per La Salute, Sezione Di Medicina Legale E Delle Assicurazioni, Università Degli Studi Di Milano, Via Luigi Mangiagalli 37, 20133 Milano, MI Italy

**Keywords:** Explosion-related death, Blast injury, Terrorism, Mass disaster, Forensic anthropology, Suicide

## Abstract

**Purpose:**

Explosion-related deaths are uncommon events which require expertise and confidence so that an appropriate death investigation can be performed. The present study aims to provide a detailed forensic analysis of the issues and implications arising in the event of an explosion.

**Methods:**

A retrospective review of casualty data was conducted on electronic literature databases. Cases concerning deadly explosions registered at the Milan Institute of Legal Medicine were examined and analyzed altogether.

**Results:**

Explosions may involve closed or open systems. A security assessment of the site is always necessary. Alterations of the site due to rescue procedures can occur; thus, on-site forensic investigation should be adapted to the environment. Then, a study protocol based on autopsy procedures is presented. Application of the postmortem radiology enforces forensic procedures both for the analysis of blast injuries and skeleton fractures, and for identification purposes. Blast injuries typically cause lacerations of the lungs, intestine and major vessels; moreover, hyoid fractures can be documented. Histopathology may help to define blast injuries effectively. Forensic chemistry, toxicology and ballistics provide useful investigative evidence as well as anthropology and genetics. Different forensic topics regarding explosions are discussed through five possible scenarios that forensic pathologists may come across. Scenarios include self-inflicted explosion deaths, domestic explosions, work-related explosions, terrorist events, and explosions caused by accidents involving heavy vehicles.

**Conclusion:**

The scenarios presented offer a useful instrument to avoid misinterpretations and evaluation errors. Procedural notes and technical aspects are provided to the readers, with an insight on collaboration with other forensic experts.

## Introduction

An explosion is defined as a violent and sudden fluid expansion, which determines a huge and rapid rise of pressure in the existing space (air or water) [1]. The main disruptive effects are caused by the movement of great air (or water) masses which provoke a rapid succession of compressive and decompressive waves [2]. Explosions can be triggered by airplane crashes, domestic gas leaks, fireworks and bombs [3, 4]. Chemical devices (bombs) are divided into low-order and high-order explosives on the basis of the speed of detonation: the first ones include black powder and smokeless powder, while the second ones are mainly represented by dynamite, cyclonite, trinitrotoluene, cyclotrimethylene trinitramine, ammonium nitrate-fuel oil and plastic explosives (e.g. C4, PE4, Semtex) [1, 5–7].

Factors such as the amount and composition of explosive material, environment, delivery methods or the distance between the victim and the explosive device are all important elements in defining the pattern, as well as the extent of injuries caused by an explosion [2, 8]. Barotrauma damages configure the major mechanism of wounding and mortality, as the shock waves, coming into contact with the gases of the lungs and the viscera, cause fatal blast injuries [3, 9]. Furthermore, blast waves can lead to the collapse of buildings and the combination of thermal energy and toxic substances released during the deflagration can result in a widespread multitude of very challenging scenarios for the forensic pathologist [1, 4, 10]. The pathophysiology of explosion-related deaths is complicated and subordinated to many factors: direct blast injuries, injuries generated from the perforation of the body by large fragments during the explosion, traumatic asphyxia in the event of a building collapsing, and acute distress respiratory syndrome due to chemical pneumonia and thermal injury [1, 7, 9, 11–13]. Injury profiles are unique if compared to non-blast accidents and as a consequence they have been categorized as four distinct groups, listed in Table 1.

**Table 1:** Classification of injuries caused by explosions

Classification of injury	Mechanism of injury	Type of injury
Primary	Direct interaction of the supersonic blast wave (spalling, implosion, inertia) with gas-containing and hollow organs of the body	Tympanic membrane rupture Hyoid bone fracture Blast lung, gas embolism, pneumothorax Heart avulsion, aorta laceration Blast intestine, intestinal perforation Traumatic amputations
Secondary	Direct trauma to the victim’s body caused by fragments or materials which have been energized by the explosion	Fragmentation/penetrating injuries such as blunt or sharp force injuries (depending on the nature of the objects) Comminuted fractures
Tertiary	Mass movement of the body or structures/objects against a body propelled by the blast wind	Spiral fractures 3-point bending fractures Axial loadings Traumatic amputations
Quaternary	Miscellaneous causes of injury, the remainder	Burns Crash injury Noxious gas inhalation injuries Traumatic asphyxia Psychological disorders

Many elements must be considered by forensic pathologists in the event of an explosion in order to perform a proper medicolegal death investigation. Among them, on-site investigation is essential due to the diversity of environments where an explosion-related death can occur [14–16]. Blasts are associated with severe damage. In particular, a variety of skin injuries can be observed at the autopsy: abrasions, bruises, lacerations, various degrees of burns, including charring, damage to the extremities or body disruption. Other findings are mainly represented by pulmonary injuries and injuries to the upper airways. Trachea and main bronchi might show lacerations or rupture while the lungs are often hyperinflated, hemorrhagic and marked by rib contusions. Barotrauma effects can also be detected in the gastrointestinal system: as for the lungs, there can be hemorrhagic injuries, large intestine avulsion or lacerations [10, 11, 14–18].

The present study aims to better clarify a topic that has been poorly analyzed in the forensic medicine literature. Problems and forensic implications related to blast events are highlighted. A classification of the different scenarios resulting from explosions that the forensic pathologist might face is provided and schematically summarized in Table 2.

**Table 2:** Classification of explosion-related deaths

Civilian setting	Non-terrorist events	Self-inflicted explosion deaths Domestic explosions Work-related explosions Heavy transport-related explosions
	Terrorist events	
Military setting	War-related explosions Terrorist attacks	

## Materials and methods

A retrospective review of casualty data was conducted, selecting the titles and abstracts of articles based on their relevance. In the most common electronic databases (Pubmed, Scopus, Medline and Web of Science) a literature research was carried out using the following combination of free text protocols, individually and randomly combined trough the Boolean operator “AND”: “explosion”, “injury”, “bomb”, “explosive”, “lesions”, “terrorism”, “suicide”, “fatality”, “burns”, “identification”, “disaster management”. Filters such as full-text, publication date and English language were also activated. Preference was given to recently published articles, but commonly referenced and highly regarded older publications were also included. Moreover, the bibliographies of the selected articles were reviewed for other relevant articles. The research resulted in less than 50 articles, mostly case reports, which matched the following criteria of inclusion (at least one):

- description of death scene investigations and forensic implications;
- discussion of postmortem evidence at autopsy;
- analysis of issues related to the interpretation of body injuries.

Additionally, pertinent forensic pathology handbooks and manuals [17–21], which include chapters dedicated to the description of explosion injuries, were used when appropriate. The cases concerning deadly explosions registered at the Institute of Legal Medicine of Milan were therefore examined. Subsequently, the selected studies were analyzed altogether.

## Results

Thirty-four scientific articles dealing with forensic pathology and explosions that fulfilled the inclusion criteria were included in the present research. Among these were 18 case reports [5, 13, 22–37], 11 original articles [7–9, 11, 12, 14–16, 38–40], 3 reviews [41–43], and 2 case series [44, 45]. No technical notes or extensive reviews focused on forensic evaluation of explosions and implications were found. The analysis of the literature revealed that several authors had described, mainly through case reports, the most common alterations and modifications of the crime scene and also the postmortem injuries caused by different types of explosives. As previously mentioned, the presence of forensic pathologists on the crime scene and the subsequent medicolegal evaluation in the autopsy room may provide useful information when dealing with blast events. Alterations caused by blasts lead, if approached incorrectly, to misleading interpretations and faulty investigative reconstructions.

The following paragraphs discuss the problems and the challenges posed by the crime scene, as well as of the body effects of explosion on the body. Different situations related to explosion are described, starting from the on-site forensic examination and autopsy procedures. Each of the five scenarios related to explosions is introduced by the description of a past event that occurred in Milan. Technical procedures and scenarios are grouped in the following subsections:

- on-site forensic examination;
- autopsy procedures;
- deaths by self-inflicted explosion;
- domestic explosions;
- work-related explosions;
- heavy transport-related explosions;
- terrorist events.

## Discussion

### On-site forensic examination

The on-site investigation in the event of an explosion plays a twofold purpose: on one hand it allows identification of the victims, while on the other hand it contributes to clarify the circumstances which caused the explosion. Unfortunately, the presence of the forensic pathologist is not always requested from the very beginning of the rescue procedures: this may result in a confused and chaotic on-site examination, complicating further operations of identification. Explosion-related deaths are characterized by widespread injuries; the environment in which the explosion took place represents an important element that must be carefully considered by the forensic pathologist [16, 20, 46, 47]. Firstly, the on-site examination is divided into either a closed or open system, as for the classification of mass disasters [8, 15, 46, 47].

**Fig. 1:**
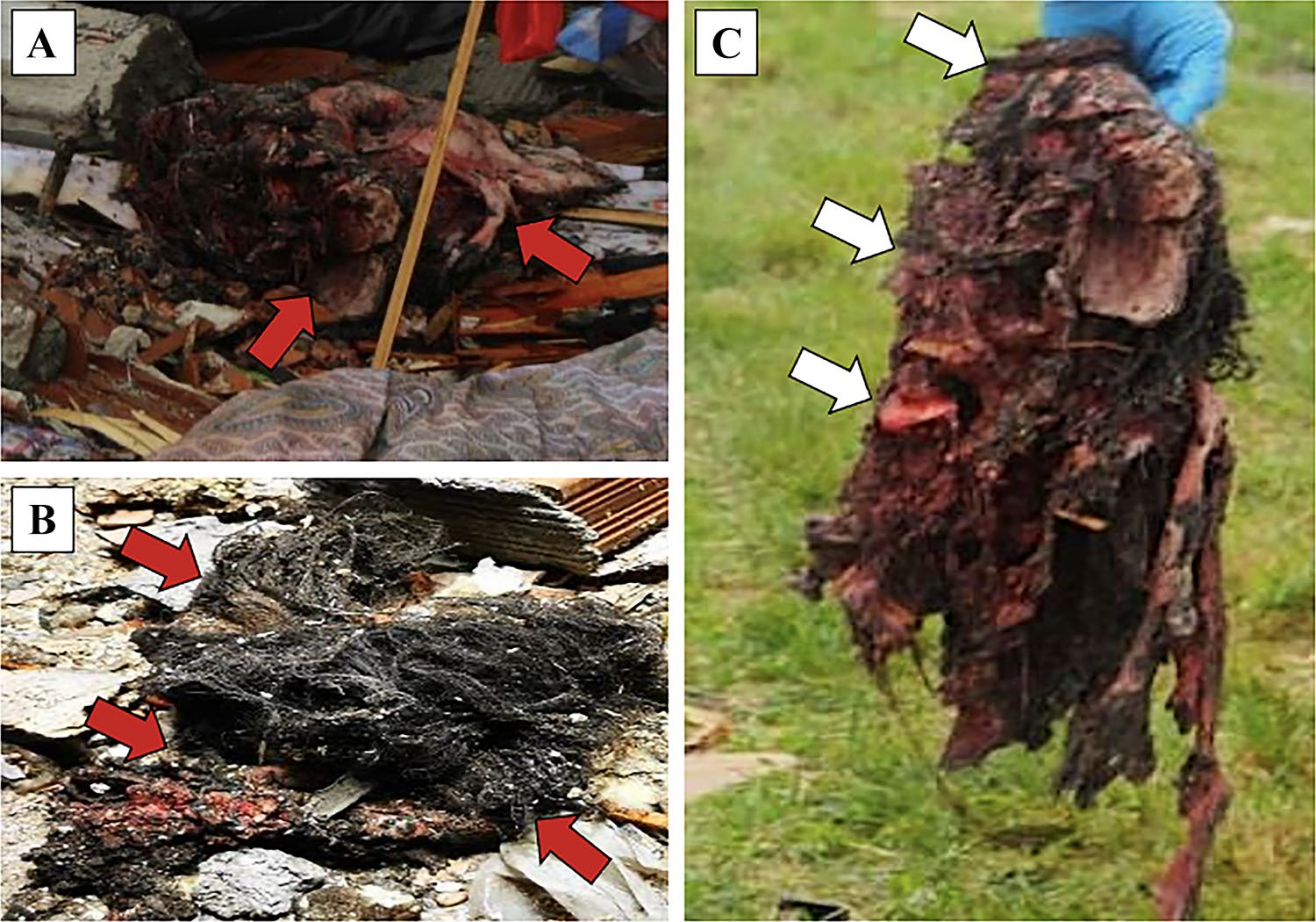
An explosion caused by ANFO (Ammonium Nitrate Fuel Oil), which occurred in a closed domestic setting. In **A** decapitation of the victim’s head (red arrows), and **B** a part of the scalp, which is completely avulsed from the face (red arrows). In **C** the face of the victim shows severe craniofacial damage, where a part of the scalp, the orbital cavities and the tongue can be still recognized (white arrows). Courtesy of Nucleo Investigativo Antincendi (NIA) Vigili del Fuoco Lombardia, Milan

**Fig. 2:**
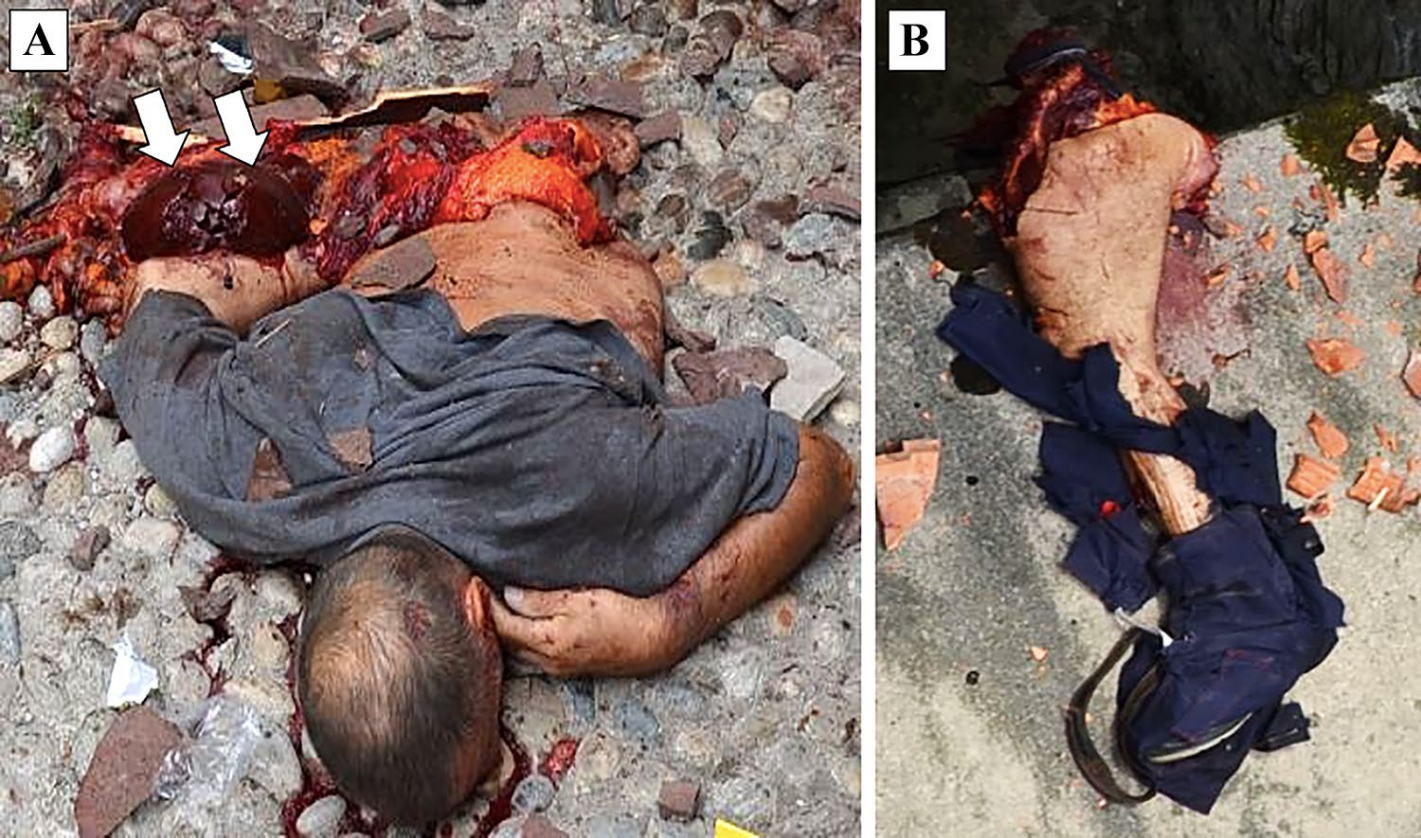
An explosion caused by LPG (liquefied petroleum gas), which occurred in an open system. The victim was working on an LPG storage tank (8,000 L), when a high-pressure gas leak hit his body. **A** The abdominal region is disrupted, with the liver lying on the ground (white arrows). **B** The left lower limb, which was found a few hundred meters away from the body. Courtesy of Nucleo Investigativo Antincendi (NIA) Vigili del Fuoco Lombardia, Milan

In a closed system the deflagration releases an amount of mechanical energy, also amplified by deflected and ricocheted pressure waves, provoking not only direct blast injuries but even the collapse of buildings such as industrial factories or houses (Fig. 1) [46]. In an open system, such as squares, the countryside or out-of-town rural areas, blast waves can project bodies or parts of them across a large area (Fig. 2), and weather conditions, local predators and the natural decay of the dead bodies can destroy important elements and hinder forensic operations [16, 20]. The whole area may be divided into smaller subareas which should be numbered in order to facilitate the recovery operations of the victims, or parts of them, including any personal items. In both cases, the explosion might result in a total obliteration of the affected area, thus preventing a thorough investigation of the scene, which is simply no longer recognizable [21, 47]. Moreover, alterations of the crime scene can occur due to damaging effects of the area, which are usually followed by rescue operations [13, 14].

As usual, during on-site investigations, the security assessment of the site should be performed before entering the area and at the same time the police should seal off the scene so that it will remain untouched and a proper investigation can take place [19, 47]. First, it is important to take as many pictures as possible (videos are even more preferable) of the scene and the surrounding area. The conditions of the site may have been deeply modified by the fire or adverse natural events. If an appropriate computer program is used, the photographs and the scanning of the scene can provide a 3D recreation of the event and thus make it possible to accentuate important details for further forensic analyses [48]. In addition, an accurate photographic record of the victims and their visible injuries, including the location of the bodies and their distance from the explosive device must be realized [20, 21].

It is important, especially for those cases of supposed atypical suicides, to give an answer to the questions asked by the local authorities so that the forensic investigations can be promptly organized. The forensic pathologist, supported by the police investigators, should obtain a detailed examination of the victim’s background: a socio-economical and personal profile, such as prior medical history, is an essential documentation for a better interpretation of the crime’s dynamics [15, 21, 22]. At the scene, the presence of any suicide note should be established, and information concerning any past suicide attempts be collected, including whether the victim possessed expertise in relation to explosives [22, 23, 44]. This investigatory approach can allow a preliminary reconstruction of the fatal event as a means of differentiating domestic accidents and homicidal explosion-related deaths from such uncommon forms of suicide.

Finally, the analysis of the scene analysis should be performed by a specialized team with competent training in rescue procedures and death scene investigations: photographing and collecting the victim’s clothes and personal items will be helpful for the following identification operations [14, 16, 38, 41, 42].

### Autopsy procedures

In the event of an explosion-related death, a study protocol based on postmortem radiology, external examination, autopsy, forensic histopathology, toxicology and anthropology is mandatory. Postmortem radiology should be the first forensic procedure performed on all bodies that have been involved in an explosion. It thereby offers the possibility to improve the observation of complex pathological findings in the autopsy room [49]. Bone and metallic fragments or other foreign objects may be easily detected at whole-body postmortem computed tomography (PMCT) [50]. Postmortem radiology may reveal small metal objects that form part of the explosive device mechanism. These may be invaluable in allowing the ballistics experts to recognize the handiwork of a particular bomb-maker or terrorist group [17]. Also, threedimensional surface scanning (3DSS) and multi-detector computed tomography (MDCT) are two techniques that are used in forensic medicine for digitalizing a body or body parts, such as bones. Interestingly, these radiological techniques allow reconstruction of severely injured skulls [51]. The instrumental approach plays two major roles in the event of an explosion: firstly, images of skeletal fractures can be evaluated whenever needed. Secondly, skull and facial digital reconstructions considerably improve identification of the victims [52, 53]. Dental radiographs are also crucial information, since they are important for dental identification, postmortem profiling, and age estimations [54–56]. Nevertheless, a complete radiographic study before the autopsy allows preservation of information for subsequent evaluations; also, it provides evidence that can guide the forensic pathologist during the autopsy.

External examination is very useful in such cases, since features of the injury patterns can be defined, and several different samples can be collected as well. Thus, forensic pathologists should collect genetic swabs, if suspicious lesions are documented on the body. Homicides may indeed be concealed by the explosion of the building where the body was set. Ballistic experts may also collect bullets, projectile fragments and evidence from explosive discharge residues from the body of the victim. Explosive traces should be analyzed by a forensic chemical expert in order to define the typology of explosive material [57]. Moreover, residual particles may be searched through the use of Scanning Electron Microscopy/Energy Dispersive X-Ray Spectroscopy (SEM/EDX), which allows different classes of particles to be defined according to their composition and images [5, 58]. Upon external examination, a thorough collection of personal items (e.g. clothes, jewels, identity cards), in association with the identification of personal descriptors such as tattoos, scars or medical devices should improve recognition of the victim. Also, the collection of fingerprints, if still available, may permit identification of the deceased.

Internal examination reveals macroscopic evidence of blast injuries. Particularly, lungs, and intestine blast injuries, major vessel lacerations and bone fractures are the most common pathological findings. Also, hyoid bone and thyroid cartilage fractures may be present. Histologic examination may help the forensic pathologist: in lungs, standard hematoxylin–eosin (HE) shows alveolar ruptures, thinning of alveolar septae, and enlargement of alveolar spaces. Other features include circumscribed subpleural, intraalveolar, and perivascular hemorrhages with a cufflike pattern. Also, Oil-Red-O (OR) histochemical staining may reveal fat embolism [12]. Furthermore, a moderate to strong hemoglobin immunoreactivity of the edema fluid within the alveolar spaces can be detected, showing a homogenous staining pattern. On the other hand, it is very important to establish the vitality of blast injuries. Thus, immunohistochemical analyses using anti-CD15, anti-IL-15 and anti-tryptase antibodies allow a precise assessment of the lesion that may have been involved in the determinism of death, representing a tool of considerable utility in explosion-related deaths [59]. Toxicological samples should also be collected; analysis may reveal toxic gases or substances inhaled or taken by the victim.

Finally, forensic pathologists should cooperate with forensic anthropologists and odontologists to improve personal identification of the victims. A dental examination carried out by a forensic odontologist is also a very important and effective method of identification, if antemortem data are available [38, 54–56]. Dental prosthesis records (fixed and removable prosthetic appliances as well) are one of the most common records used for identification purposes. Forensic odontologists may also analyze morphological features of extraoral and intraoral structures such as lip print patterns and rugae patterns, which are important elements for recognition of victims [60]. Genetic analysis on muscle or bone samples of the victims may confirm identity, if any relative is available.

**Fig. 3:**
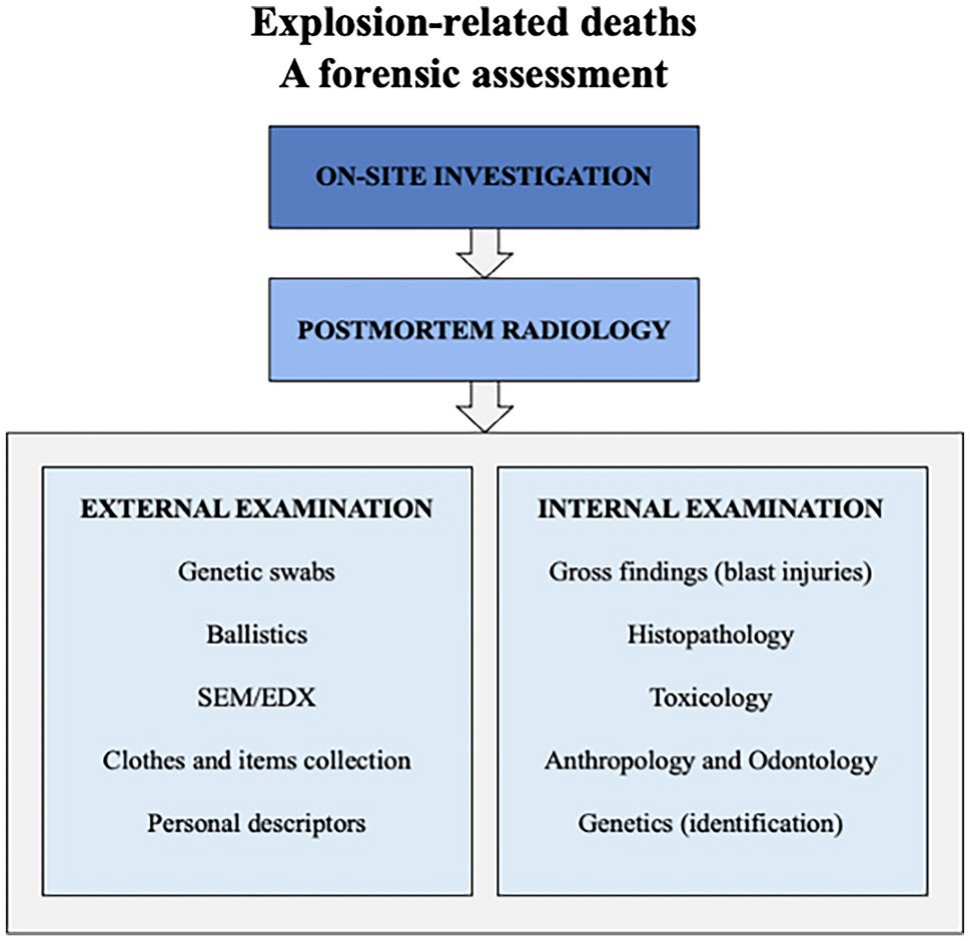
A forensic assessment to explosion-related deaths

A schematic forensic assessment in the event of an explosion is shown in Fig. 3.

### Self-inflicted explosion deaths

*In September 2006, an explosion occurred in an apartment building on the outskirts of Milan. The building collapsed and 4 people died. After the conclusion of the investigations, the cause of the explosive event was established as a gas leak provoked by a woman with suicidal intent.*

Suicide by using an explosive device, when not involved in a terrorist attack, is quite uncommon in medicolegal practice when compared with other routinely adopted methods [23, 44]. The case presented is a further exception, since the possibility to mistake this suicidal event with an accident or homicide relies mainly on circumstantial findings. In the literature of the last thirty years, a few case reports [7, 24–35] and one older study from Sweden [39] about self-inflicted explosion deaths have been published. In the cases reported [7, 23–36, 39], victims were men, ranging between 20 and 76 years-old (mean 37.7 years), and except for one case (with no risk factors described) [24], all suffered from psychiatric diseases, alcoholism, drug addiction, financial or personal problems [22–36, 39, 44].

In the analysis performed, three different patterns of non-terrorist explosion-related suicides can be defined, as follows:

- The first pattern, which is the most frequent, shows a specific involvement of the cephalic region due to placement and detonation of fireworks or other explosive materials directly on the head or inside the oral cavity [22–31]. At autopsy, the injuries typically show a regular and symmetrical pattern, primarily consisting of severe craniofacial damages such as lacerations and blunt trauma of the lips, gums, palate, nose and orbits associated with extensive comminuted fractures of the mandible, maxilla and base of the cranium [22, 26, 44]. In addition, trauma can provoke deep cortical lacerations or pulpification of brain matter, albeit decapitation or complete head destruction have been rarely described [24, 25]. Burns and gross tissue damages to the upper limbs, and, in particular, of the hand used for triggering the explosive device, provide possible evidence for suicide [27–29]. Other findings may include soft tissue and muscular hemorrhage of the neck, fracture and dislocation of the hyoid bone, lacerations and bruises of the lungs and the avulsion of the heart as a consequence of the direct effect of blast waves [22–24].
- The second pattern is identified in victims who clutch an explosive device in close proximity to the abdominal region. The injury pattern depends on the blast magnitude of the device. Siciliano et al. described the case of a 32-year-old man whose suicide was due to deflagration of a hand grenade which divided the body into two parts [32]. Similarly, Varga and Csabai reported the case of a 2v3-year-old man who located at the right side of the abdomen a homemade explosive device (a mixture of potassium cholate, sulfur and antimony) which caused the destruction of the heart, liver, diaphragm and lungs [33]. Tsokos et al. published the suicide of a 39-year-old man where death occurred in a combination of craniocerebral, abdominal and pelvic trauma with rupture of the large vessels as a result of a homemade bomb explosion [44].
- The third pattern involves cases which do not match the two patterns previously described. Particularly, an unusual suicidal explosive death of a 55-year-old male induced by intentional methane gas leak inside his bungalow was reported [34]; also, the case of 30-year-old man who was found dead in a truck cabin after the detonation and explosion of an acetylene cylinder [35]. In the first case, the cause of death was attributed to massive pulmonary fat embolism, extensive gastrointestinal epithelial burns, and acute respiratory distress syndrome (ARDS); while the second one resulted in charring and severe blast lung injuries.

Since in these cases victims who committed suicide by this uncommon manner were males, great caution should be paid by the forensic pathologist when dealing with a case of a woman suspected of a self-inflicted explosion death. A suspicion of murder or accident should be kept in consideration, until proven otherwise.

Further considerations are required in the evaluations of the damage patterns related to explosions, considering that such injuries can easily be mistaken for shotgun wounds. Hence, the absence of a firearm and the absence of any bullets at the scene, but the presence of radiopaque fragments within the body at the postmortem radiological examination, should direct suspicion towards the use of explosive devices [22, 42, 44]. Finally, an accurate assessment of the victim’s background must be conducted in order to determine the manner of death: look for possible access to explosive materials, psychiatric disorders or any personal problems of the deceased [13, 26, 29, 61].

### Domestic explosions

*In June 2016, a blast occurred in an apartment building in the south of Milan. After the explosion, the building collapsed, and 3 people died. Investigations identified the cause of the explosive event as a methane gas leak caused by a male saboteur with homicidal intentions.*

This subcategory of explosion-related deaths includes fatalities by explosion that occurred in a domestic setting. Among these, accidental gas leak represents a common event worldwide [1, 4, 6, 7, 11, 17–19, 46]. The scene is characterized by building collapse, fire, debris and a cloud of dust, which make rescue operations protracted and very difficult.

**Fig. 4:**
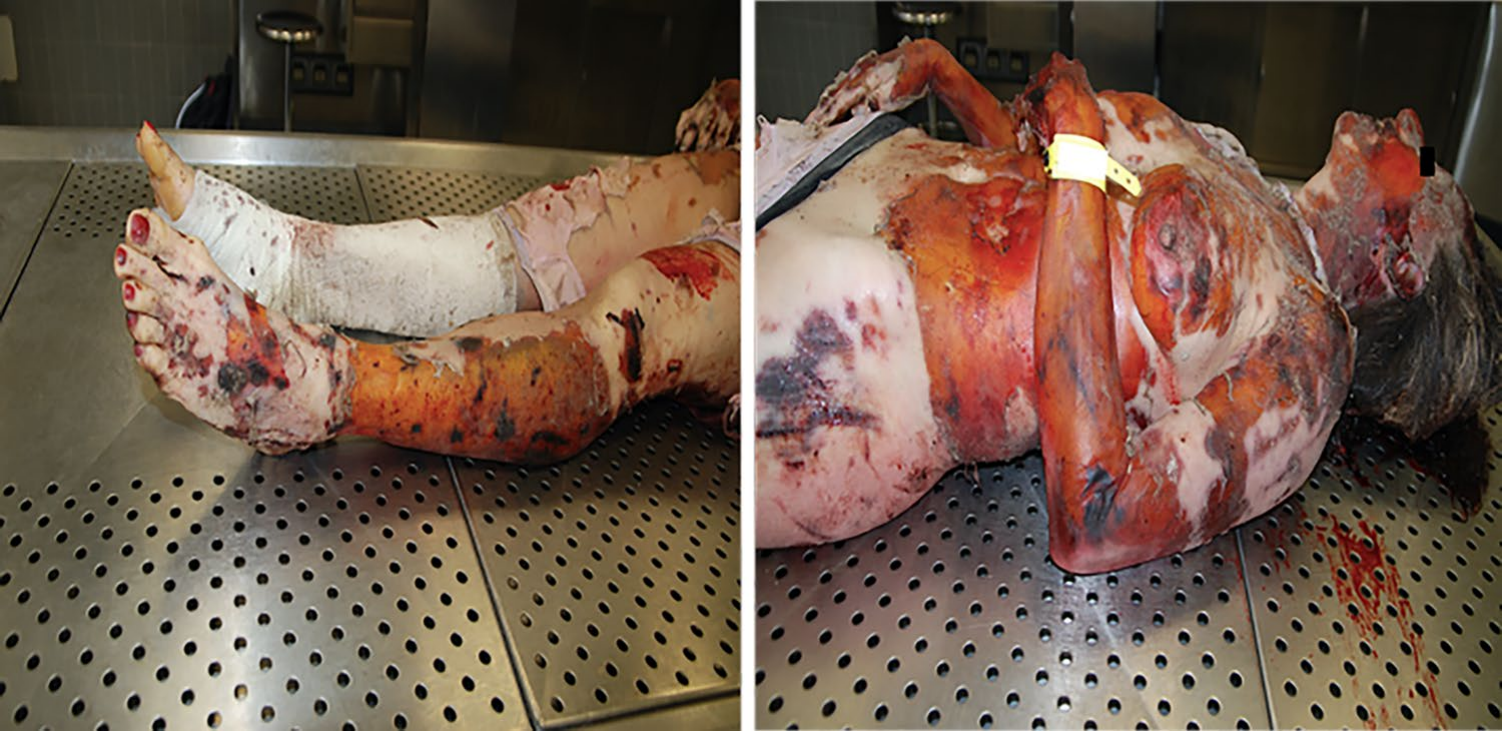
Bruises, abrasions, and different degrees of burns on the victim’s body; furthermore, dust can be seen on the face and the hair of the victim. The woman was involved in a domestic explosion, which caused the collapse of a building

**Fig. 5:**
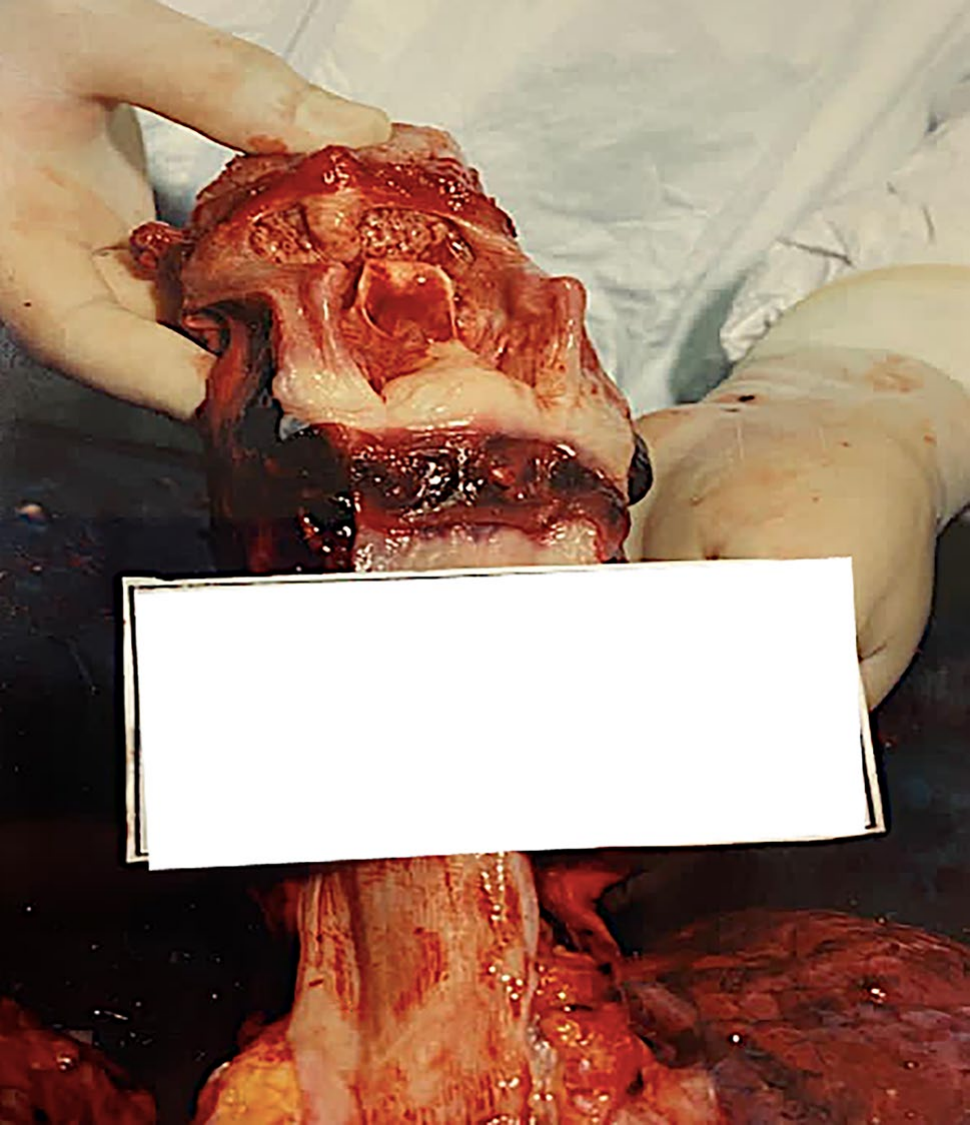
Blast effects on the larynx resulting in a deep laceration of the thyroid and cricoid cartilages. This injury was caused by a domestic explosion related to a gas leak

At autopsy, external examination can reveal a diffusely edematous body with multiple wounds [34], mainly lacerations, many abrasions along with different degrees of burns (Fig. 4). Internal examination usually shows pulmonary injuries and injuries to the upper airways: the trachea and main bronchi are lacerated while the lungs are often hyperinflated with severe blunt-force trauma and lacerations [1, 4]. Also, traumatic asphyxia can frequently lead to death due to the massive amount of dust inhaled in the event of building collapse or to fixation of the chest by rubble [17–19]. The abdomen can be heavily damaged with organs torn off from the abdominal wall, such as the stomach, intestines and kidneys avulsion or hepatic and splenic gross lacerations [1, 17–19]. An additional finding is the fracture of the thyroid cartilage (Fig. 5), which is produced by the explosive barotrauma or direct violent impact. When this evidence is found, attention must be paid to avoid misinterpretation regarding homicidal strangulation [11, 17, 22]. Autopsy helps to confirm the cause of death of the victims; also, it contributes to the assessment of the manner of death. Hence, a close cooperation between the forensic pathologists and the investigators can differentiate sabotage from accidents in cases of domestic explosive-related deaths.

### Work-related explosions

*In November 2010, an explosion occurred in the industrial area of a small town close to the metropolitan city of Milan. The point of blast origin was the storage site for hazardous waste due to a malfunction of safety systems. 4 workers died.*

Work-related fatalities are a common phenomenon worldwide. Hazardous work is represented by coal mines (especially in the past for firedamp leaks), chemical industrial sites or firecracker factories, where explosions can typically occur [7, 36, 39]. In the literature, a few cases have been reported such as explosions caused by coffee-making machines, autoclave machines, or while repairing an air conditioner compressor [45]. A common pattern can be identified in the evidence that explosions derived from high-pressure appliances [7, 40, 45].

**Fig. 6:**
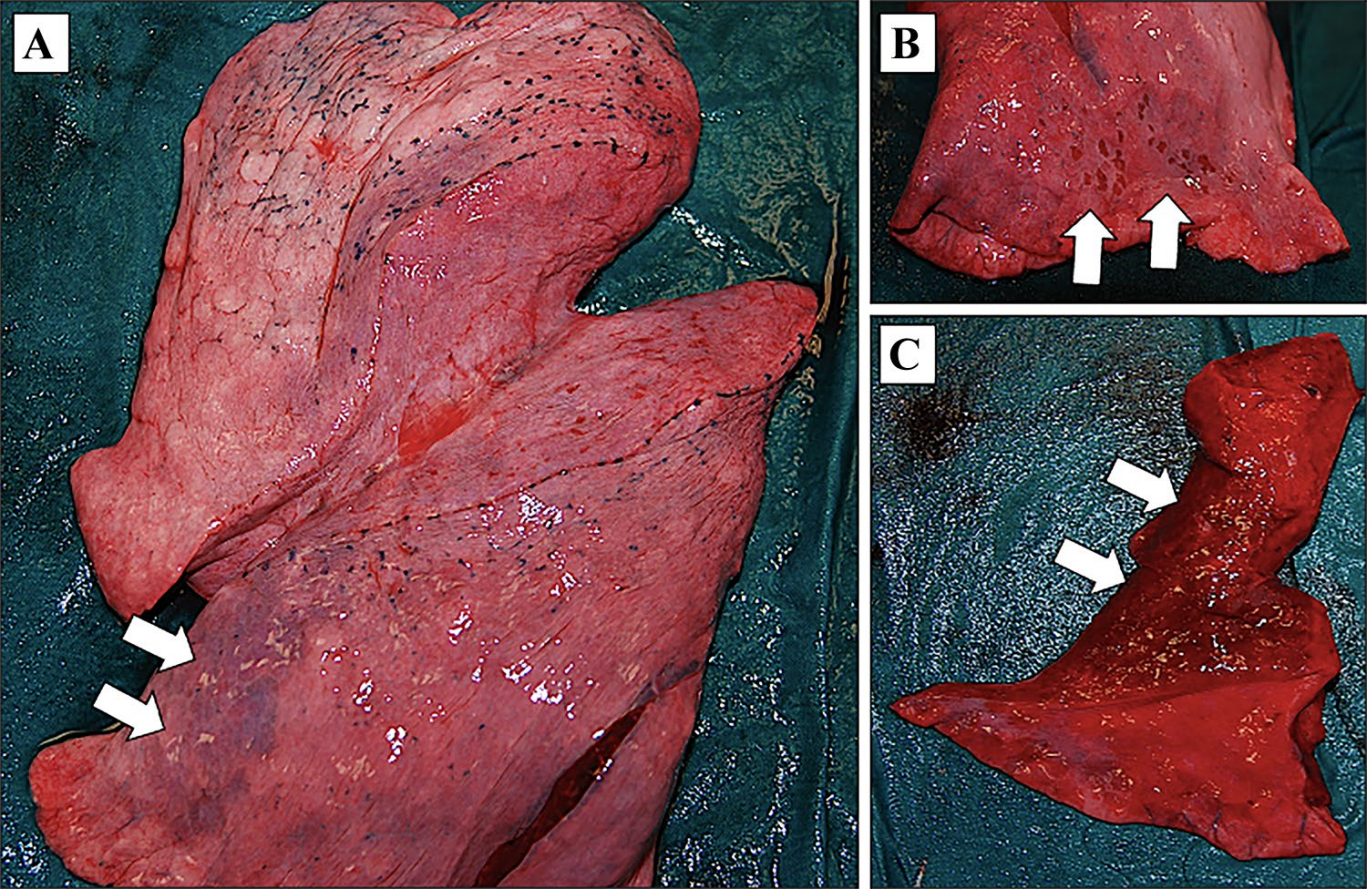
Blast effects on the lung. **A** The left lung is hyperinflated with pulmonary contusions (white arrows); **B** Subpleural patchy hemorrhages on the diaphragm surface of the lung (white arrows); **C** The pulmonary parenchyma is markedly hemorrhagic (white arrows)

Byard described the risk of work-related explosions in the enclosed engine room of fishing vessels if volatilized fuel is not adequately removed. The flammable properties of the mixture are determined by the ratio of fuel vapor to air: the speed and magnitude of combustion can be extremely violent, resulting in an explosion [43]. Furthermore, an unusual “work-related” death occurred in Milan in 2017, when a thief died while attempting to break into an automated teller machine (ATM) using the explosive power of an acetylene gas cylinder [37]. Most of the cases were accidental events; moreover, victims frequently died after the explosive accident or within the first 24 h, reflecting the seriousness of the injuries reported by the workers [40]. The injury pattern showed a combination of blast effects, burns, and flying missile injuries which damaged internal organs. The lungs may also show subpleural patchy hemorrhages, pulmonary contusions and intrapulmonary hemorrhage (Fig. 6). The predominant site of lethal wounding was the head, followed by the chest or the combination of both [36, 37, 40, 43, 45].

In cases like the ones mentioned above, forensic examination of the dead bodies revealed mainly several burn injuries: different degrees of burns are differently distributed all over the body, including significant charring. Charred and extensively damaged bodies require a careful examination for identification purposes [20, 21, 41]. Nowadays, workplace deaths represent an important social problem [40]: the reconstruction of the event allows authorities to find out which responsibilities involved the employer and which ones the worker.

### Heavy transport-related explosions

*In October 2001, two aircrafts collided on the runway at Milan city airport “Linate”. The crash occurred in weather conditions of heavy fog and was caused by nonfunctioning and nonconforming safety systems. After the collision, a fire explosion was produced, resulting in 118 deaths and no survivors.*

A variety of events can lead to explosions related to large transport vehicles. Aircraft accidents, such as the example reported above, railway disasters, maritime accidents and other mass disasters can be included in this category [20]. In these cases, the explosion produces a significant kinetic effect as a result of collisions between objects travelling at high speed [19]. Since the mechanical shock wave cannot dissipate the energy of the impact itself, the accumulation of heat and pressure at the contact site instantly breaks the two objects, projecting their fragments in every direction and vaporizing or partially melting the material [7, 38]. These events result in a wide number of fatalities, the remains of which, being often severely traumatized, charred and/or dismembered, need to be identified and subjected to medicolegal investigation [1, 2, 13, 20].

Two major factors complicate the recovery of human remains: body destruction and context [40]. Since explosion may lead to extreme body fragmentation, care must be taken in analysis at the scene [15, 19, 47]. Forensic analysis of remains must include assessment of minimum number of individuals and commingling issues as well as identification [17, 18, 54, 55, 56]. Identifications are frequently facilitated by recovery and analysis of identification tags, dental restorations and surgical materials [41]. The context is important since in most cases human remains are located in proximity to other materials of similar appearance [20, 21]. Ideally, forensic anthropologists should participate in the recovery since they are experts in recognizing fragmented human remains [17, 41]. In the case mentioned above, the team of experts guided by forensic odontologists succeed identifying all the victims, through consultation of the victims’ dental records, a fundamental part of the entire investigation [38, 60].

At autopsy, trauma interpretation can be difficult due to the multiplicity of skeletal and visceral injuries with a prevalence of heat-related lesions, usually complete charring and fragmentation [41]. The distinction of different injury patterns may provide important information for the investigators, since one of the priorities is to reconstruct the dynamic of the event to differentiate accidental events from sabotage.

### Terrorist events

*In December 1969, during a terrorist attack, a dynamite bomb exploded at the headquarters of Banca Nazionale dell’Agricoltura, a few hundred meters from the Duomo of Milan. 17 people were killed and 88 wounded. On the same day, a second unexploded bomb was found.*

In cases of explosive military devices, blast effects uncommonly lead to death since the terrorist bomb usually produces low energy barotrauma in close proximity to the explosion. For more powerful bombs, flash burns can occur on individuals nearby [2, 3].

**Fig. 7:**
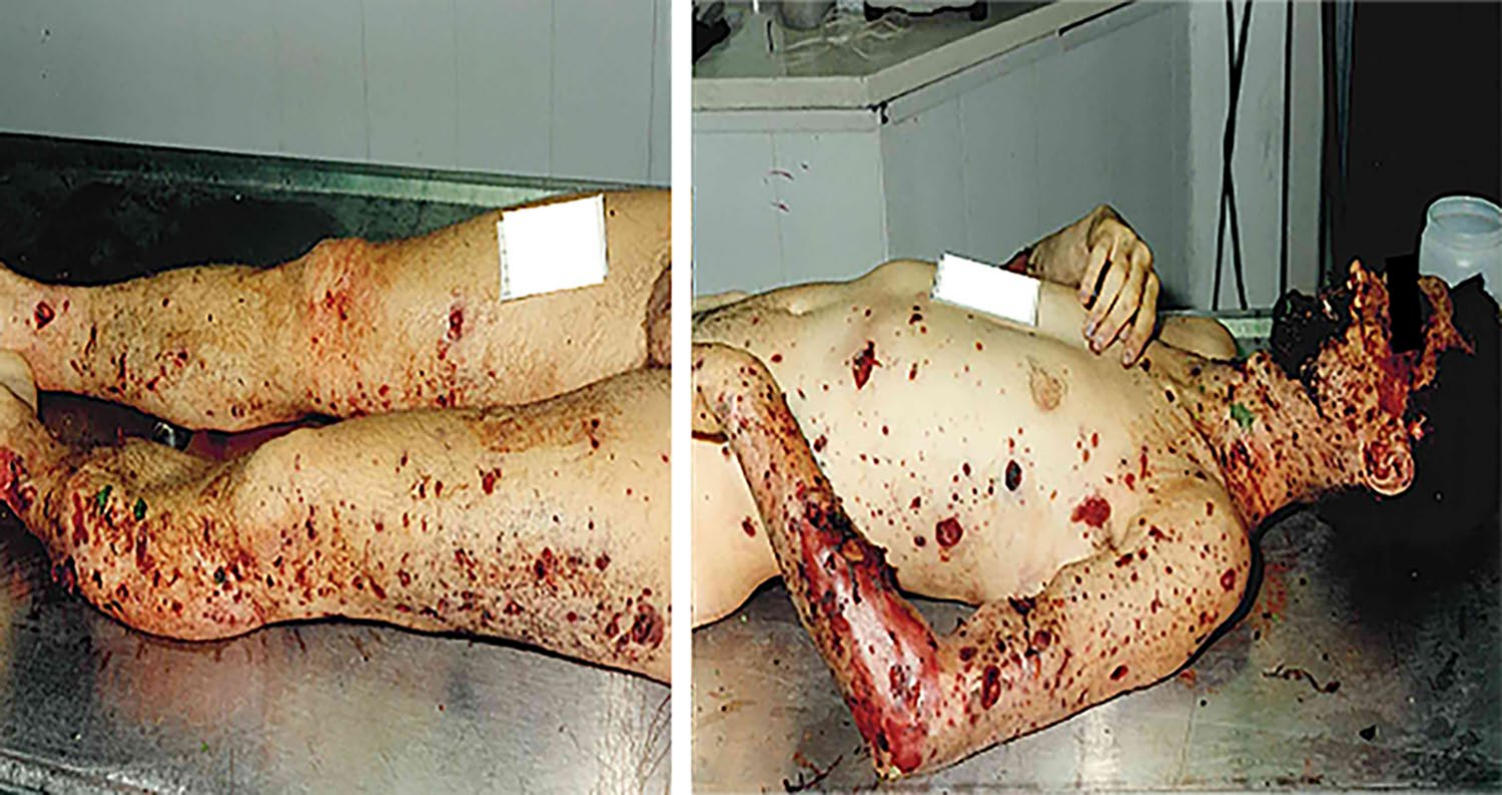
Peppered appearance of the skin caused by the small missiles creating the typical triad of bruises, abrasions and lacerations. These injuries were caused by an explosive device (trinitrotoluene), which was hidden in a car

**Fig. 8:**
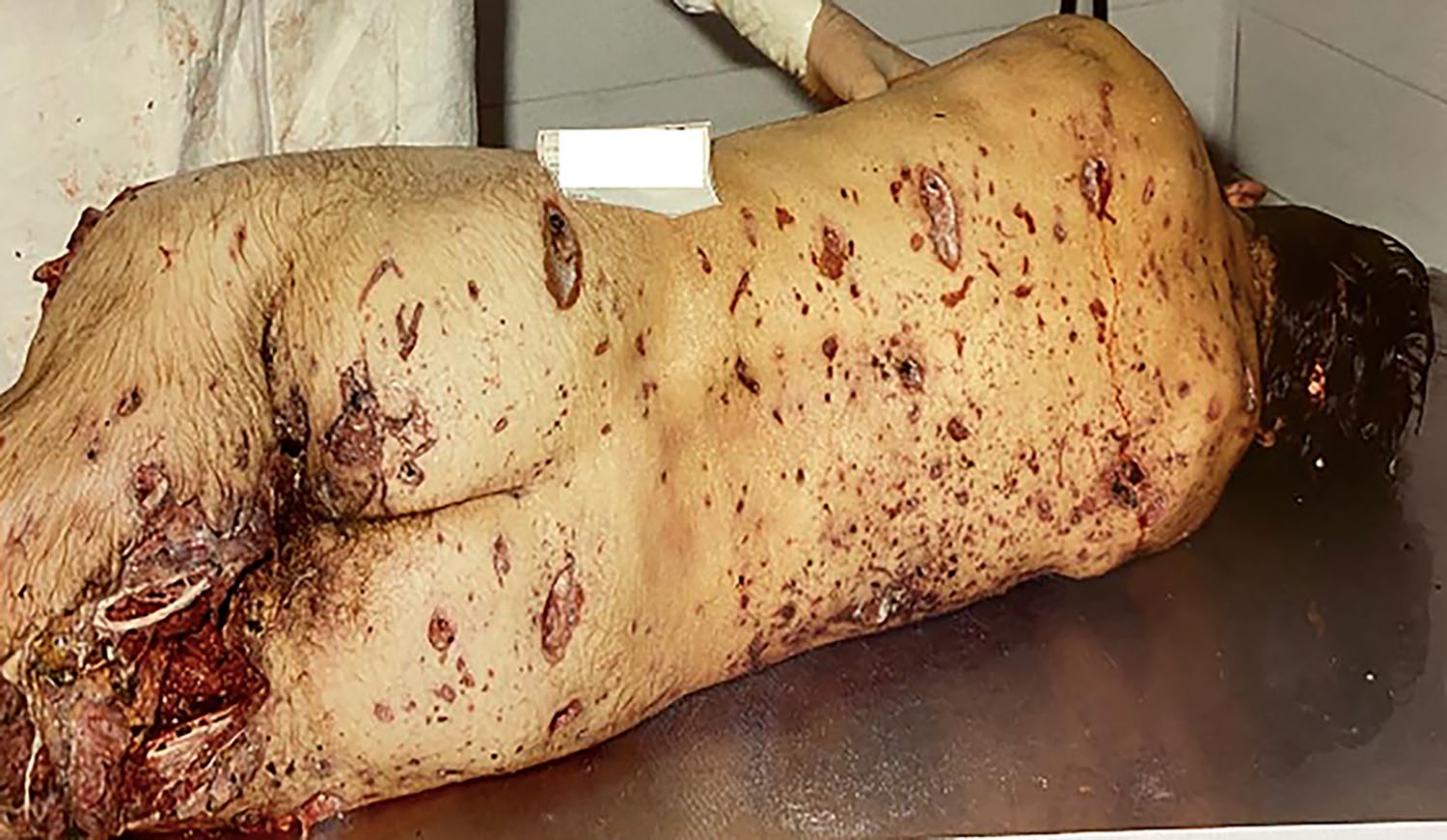
Peppered appearance of the skin with the lower extremities partially disrupted. These injuries were caused by an explosive device (trinitrotoluene), which was hidden in a car

The individual in contact with or near the seat of explosion can be blown to pieces and scattered by the force of explosion gases [8]. Solid fragments from the device, such as the case or the conveyance, in which the bomb was concealed (a car usually is usually the most common vehicle used), can be projected at high speed and over a wide area, hitting the bodies of people nearby [2]. Metallic pieces can act like bullets, causing significant or fatal injuries [13, 18]. In these cases, the autopsy shows a multiplicity of injuries such as a pigmented dust produced by the explosion on skin and clothing, the burning of hair and eyebrows, and the laceration and ignition of clothing [10, 21]. Also, the so-called “peppering” appearance of the skin caused by the small missiles creating the typical triad of lesions (bruises, abrasions and lacerations) can be assessed (Fig. 7) [9, 17]. The body can be totally or partially destroyed: extremities are equally involved resulting in hands, arms, feet and legs disrupted or ripped out (Fig. 8). Localized injuries at the legs and the abdominal region have been assessed on the body of the terrorist when a premature deflagration occurs [8, 14, 42]; it can be an important element to be considered in order to distinguish between bombers and suicidal attacks. The number and distribution of fractures can help the differentiation of injury mechanisms, as they are relevant criteria for distinguishing an explosive charge carrier from a victim [13, 18, 42]. According to the literature, multiple injuries resulted in the cause of death, followed by localized injuries at the head, then the chest and the abdomen [6, 8, 14].

Various ballistic and forensic experts may be involved in terrorist events; among them, chemists and engineers will have the task of determining the composition and position of the device. The forensic pathologist can be part of the event reconstruction, as localized severe trauma indicates the relative position of the bomb and the victim at the time of detonation [14]. Trace evidence of explosive residues can be found on the bodies and further analyzed [5]. In this way, investigations can receive a fundamental support in identifying also the bomb-carrier and, thus, the precise dynamics of the event [17, 18, 42]. In the case mentioned above, starting from the autopsy reports, the team of experts in charge managed to develop a visual record placing each victim in the most probable position at the time of the explosion, playing a pivotal role in the forensic proceedings.

## Conclusions

Explosion-related deaths are a critical issue for forensic pathologists since a proper investigative reconstruction and interpretation are based on many different pieces of information. Hence, this review shows that death caused by explosions is an uncommon event, with the majority of cases occurring in the workplace and in a domestic setting. Non-terrorist self-inflicted deaths are exceptional, especially if they are associated with the detonation of the explosive in close proximity to the head and abdomen. Non-terrorist homicides using explosive devices are even less common. Moreover, it is important to be aware that the integrity of both the scene and the victim’s body may be damaged by alterations due to human and environmental factors.

Once on-site forensic examination is completed, forensic pathology investigations should therefore rely on study protocols based on precise procedures for postmortem analysis. Postmortem radiology allows the preservation of information for subsequent evaluations; also, it provides evidence that can guide the forensic pathologist during the autopsy. This approach improves the analysis of complex pathological findings of blast injuries. Moreover, forensic histopathology, toxicology, genetics, chemistry, and anthropology are key elements to achieve successful outcomes. The correct training for a forensic pathologist should include expertise regarding death produced by blasts, preferably through crime scene simulations to avoid misinterpretations and evaluation errors. Forensic and emergency teams must know the patterns of the injuries caused by explosives. Ideally, a proper and structured postmortem assessment in explosion-related cases must observe the phases summarized as follows:

- on-site forensic investigation;
- postmortem radiological examination;
- external examination to gather surface evidence (genetics, chemistry, ballistics), collect clothes and items, and note personal descriptors and document traumatic injuries;
- internal examination to analyze both macroscopic pathological findings of blast injuries and other traumatic lesions; also, the autopsy allows samples to be collected for histopathological, toxicological, anthropological, odontological and genetic analyses.

In conclusion, a multi-disciplinary approach is highly recommended in death investigations. Especially, when dealing with explosion-related deaths, a close cooperation between forensic pathologists and other forensic professionals, such as anthropologists, radiologists, engineers, ballistic experts, and chemists is preferable, in order to provide a high-quality forensic investigation and consequent evaluation.

## Key points

- 1. Forensic pathologists may deal with different and complex explosion-related scenarios.
- 2. For blast injuries, postmortem radiology before autopsy examination is mandatory.
- 3. Identification of explosive residue particles requires ballistic and chemistry experts.
- 4. Blast injuries typically involve the lungs, intestine, and major vessels.
- 5. Forensic anthropologists and odontologists may help the pathologist to identify victims of explosions.
